# Phosphorus partitioning contribute to phosphorus use efficiency during grain filling in *Zea mays*

**DOI:** 10.3389/fpls.2023.1223532

**Published:** 2023-07-04

**Authors:** Yan Sun, Yang Han, Zikai Xu, Jinting Zhang, Jianbo Shen, Lingyun Cheng

**Affiliations:** College of Resources and Environmental Sciences, National Academy of Agriculture Green Development, Key Laboratory of Plant–Soil Interactions, Ministry of Education, State Key Laboratory of Nutrient Use and Management (SKL-NUM), China Agricultural University, Beijing, China

**Keywords:** post-silking phosphorus uptake, phosphorus remobilization, grain formation, grain phosphorus, human nutrition and health

## Abstract

**Introduction:**

Lower phosphorus (P) availability limits crop productivity in agroecosystems. The remobilization of P from the source to the sink organs plays an important role in enhancing the P-utilization efficiency of crops. During the grain filling stage, phosphorus flow to the developing grains, the primary sink, determines crop yield. However, the specific contributions of different organs to grain P throughout the post-silking period in maize remain unclear.

**Methods:**

In our study, three maize inbred lines (CIMBL89, Ji846, and CML118) with contrasting P statuses were selected and grown in a field with high P (HP, 150 kg ha–1 P2O5) and low P (LP, 0 kg ha–1 P2O5) conditions.

**Results:**

The grain yield of CIMBL89 was 69% and 169% greater under HP supply, and 83% and 309% greater than those of Ji846 and CML118 under LP supply, respectively. The ear length, ear diameter, and kernel row number of CML118 were lower than those of CIMBL89 and Ji846 under HP conditions. Most of the P (87%) in the grains of CIMBL89 came from P uptake at the LP supply, while almost all P (95%) came from P remobilization in various organs at the HP supply after silking. In contrast, 91% of the P found in the grain of CML118 came from P remobilization under LP supply, while 76% came from P uptake under HP supply after silking.

**Discussion:**

In conclusion, our findings suggest that CIMBL89, with greater P acquisition efficiency, contributes to grain formation and production during the post-silking period under LP conditions. Additionally, CIMBL89 can fully remobilize P and avoid the extravagant absorption of P in P-sufficient soil, which sets it apart from Ji846 and CML118.

## 1 Introduction

Phosphorus (P) is an indispensable macronutrient required for crop growth and development, and is a component of numerous chemical molecules (e.g., nucleic acids, phospholipids, and adenosine triphosphate) and a catalyst for various biological reactions (e.g., such as energy transfer and protein activation) (Cruz-Ramírez et al., 2006; Raboy, 2009; Hawkesford et al., 2012). Phosphorus is mainly taken up by plants in the form of inorganic P. However, the poor mobility and bioavailability of inorganic P in soil is due to its high binding to organic and inorganic compounds or its low solubility in sparsely soluble minerals, which tends to reduce the diffusion capacity (López-Arredondo et al., 2014; Pant et al., 2015). Lower P availability in farmlands largely limits crop productivity (Cruz-Ramírez et al., 2006). The three main drivers affecting P flow from farmlands are P fertilizers, post-harvest losses of agricultural products, and P losses due to agricultural runoff (Kumar et al., 2021). Phosphorus fertilizer application is the most direct cultural intervention to replace P nutrient loss in multiple cases, helping to improve yields and soil fertility (Cordell et al., 2009). Phosphorus fertilizer is derived from phosphate rock, a non-renewable resource with a limited and unequal distribution in the Earth’s crust (Vance et al., 2003; Cordell et al., 2009). The law of demand and supply of phosphate rock is reflected, and it is estimated that global phosphate rock will be exhausted by 2040 (Martin et al., 2019). As a result, humans are facing serious physical environmental problems caused by the loss of unused P from intensive agricultural production, while facing dwindling phosphate rock resources and reserves (Baker et al., 2015). This is a serious deviation from the desired trajectory of achieving global food security and sustainable production systems, and is not conducive to promoting green agricultural development and modernizing agriculture. Over the past few decades, scientific researchers have focused on improving the P-acquisition efficiency (PAE) and P-utilization efficiency (PUE) of crops. Breeding crops with high P efficiency, low P tolerance, and high yield traits will have an impact on crop yield and reduce P fertilizer inputs, minimizing possible environmental risks (Hammond et al., 2009; White et al., 2013; Dissanayaka et al., 2018; Lynch, 2019; Wang et al., 2019a).

The transition from vegetative growth to reproductive development phase, external P uptake, and internal P remobilization change gradually with time, especially during the grain-filling period of cereal crops, which is the most critical period for crop yield formation (Yang and Zhang, 2006; Veneklaas et al., 2012). The yield potential of crops is closely related to the source-sink relationship, and the internal remobilization of P from various source organs and sink strength is an important research topic (Yu et al., 2015). The strategy for coping with lower P availability at the seedling stage relies mainly on P transfer from older and fully expanded leaves to ensure plant survival, as roots cannot obtain sufficient P from the external environment (Hill, 1980; Veneklaas et al., 2012). In rice (*Oryza sativa*), *OsPHT1;3* is specifically expressed in the phloem of the vascular tissue of the basal nodes and is responsible for the transportation of phosphate (Pi) from mature leaves to young leaves (Chang et al., 2019). In addition, the Pi transporter *OsPHT1;8* plays an important role in the distribution of Pi from the source organs to sink organs (Li et al., 2015). *ZmPT7*, a close homologue of *OsPHT1;8*, is mainly expressed in mature leaves and is involved in the distribution of Pi from mature leaves to young leaves, and P content was significantly lower in older leaves of the *ZmPT7*-overexpression lines compared with wild-type plants (Wang et al., 2020b). *OsPHT1;7* is preferentially expressed in source leaves and nodes and is an important Pi transporter in rice. *ospht1;7* mutant plants showed increased Pi content in source tissues and decreased Pi content in sink tissues (Dai et al., 2022). The high transcript abundance of *OsPHT1;7* in anthers, accumulation of Pi in anthers, and germination of pollen grains were affected, which eventually led to crop yield reduction (Dai et al., 2022). The remobilization of Pi from vegetative tissues to grains during grain filling is an important part of the global P cycle (Jeong et al., 2017). *HvSPDT* (*SULTR-like phosphorus distribution transporter*), a node-localized transporter in barley (*Hordeum vulgare*), is involved in the distribution of P into grains, especially under Pi deficiency. Knockout of *HvSPDT* significantly reduced grain P and caused a severe yield reduction (Gu et al., 2022). Similarly, knockout of *OsSPDT* reduced P accumulation in rice grains without yield and seed vigor penalties (Yamaji et al., 2017).

For cereal crops such as wheat (*Triticum aestivum*), maize (*Zea mays*), rice, and barley, grains are the main P pool in the later growth period. Approximately 60%–85% of total plant P is eventually allocated to the grains to supply their growth and development, and the major form of P storage is phytic acid (PA) (Raboy, 2001; Dissanayaka et al., 2018). However, since humans and non-ruminant animals such as pigs, fish, and poultry cannot produce enough phytase to effectively release the phytate groups in PA, they cannot be digested, absorbed, and utilized, and then enter rivers, lakes, and oceans with manure, leading to a series of negative environmental problems, such as eutrophication of water bodies (Raboy, 2009). In recent years, there has been growing concern for food safety and nutritional health, along with the continuous improvement of material living standards and spiritual pursuits. Therefore, ensuring that the edible parts of cereal crops (mainly grains) are rich in nutrients while stabilizing crop yields is crucial for achieving the dual sustainable development of food yield and nutritional security (Yu and Tian, 2018). The development of the three green revolutions has led to high yields of cereal crops, but the scale of crop production has expanded. As a result, nutritional deficiencies and insecurity have become a major challenge in the process of human civilization. This food-based approach to agricultural development is considered a strategic tool to combat malnutrition and achieve sustainable agricultural development (FAO, 2014). Heavy reliance on cereal-based diets constitutes a significant global issue arising from micronutrient malnutrition with lifelong implications for human health (Kennedy et al., 2003). Biofortification interventions, such as crop breeding and fertilizer applications, appear to be an effective strategy for enhancing the bioavailability of micronutrients in cereal crops, such as maize, wheat, and rice (Welch and Graham, 2004; White and Broadley, 2005; Cakmak, 2008). For most genotypes, grain P concentrations above 0.9 mg g^−1^ in rice are sufficient to ensure high seed germination and vigorous seedling growth, except for varieties sensitive to reduced grain P concentrations at the seedling stage (Pariasca-Tanaka et al., 2015). The higher the level of excess P in the form of PA, the lower the PUE, resulting in a high crop demand for P fertilizers (Shenoy and Kalagudi, 2005). That is, reducing grain P concentrations without disturbing seed germination and seedling growth vigor may not affect crop yield but will improve the sustainability of existing P fertilizer utilization.

However, during the grain formation and filling stages, the two possible sources of grain P (root acquisition or P remobilization from vegetative organs to grains) (Wang et al., 2016) and the proportion of P contributed by different vegetative organs remain unclear. In the present study, three maize inbred lines (CIMBL89, Ji846, and CML118) with contrasting P statuses were screened to investigate post-silking P uptake, remobilization, and the specific contribution of P in different organ fluxes to grain P. Additionally, we estimated the phytic acid concentration and Zn and Fe bioavailability in the maize grains. Overall, these results lay the foundation for promoting the dual sustainable development of P utilization and nutritional security, which is crucial for the well-being of both humans and the environment.

## 2 Materials and methods

### 2.1 Plant material and experiment design

In this study, three inbred maize lines (CIMBL89, Ji846, and CML118) with contrasting P concentrations were used (**Supplemental Figure 1**; **Table 1**), which were located at the Shangzhuang Experimental Station of China Agricultural University (Beijing, BJ, 116°11′ E, 40°08′ N) in spring 2021. The region has a semi-humid monsoon climate with seasonal temperatures characterized by an average annual air temperature of 10–12 °C, an average annual precipitation of approximately 600–650 mm, an annual sunlight duration of 2,700–2,800 h, and a frost-free period of 180–200 d. The soil at the study site is classified as a silty sandy loam, the soil (0–30 cm layer) with pH (1:5 soil to water) 8.1, organic carbon 8.4 g kg^−1^, total nitrogen (N) 0.78 g kg^−1^, Olsen P 6.9 mg kg^−1^, available potassium (K) 84 mg kg^−1^, soil bulk density 1.44 g cm^−3^. Two plots measuring 9 × 8 m each were treated with low P (LP) and high P (HP). A randomized block design with four replicates was used for each plot and three genotypes were planted. Fertilizer (N and K_2_O) application per hectare was 225 kg N (urea, 46%) and 80 kg K_2_O (potassium sulfate, 50%). In addition, 150 kg of P_2_O_5_ (super phosphate, 18%) should be applied under HP treatment but not under LP treatment. Two coated maize seeds were manually sown in each small hole (approximately 2 inches) with 60 cm and 22.5 cm, spacing between maize intraspecific rows and interspecific rows, respectively. Conventional agronomic measures, including irrigation and pest and weed control, were used for field management, unless otherwise stated. During the silking period, bagging was performed reasonably, and each plant was artificially self-pollinated three to four times.

**Table 1:** Detailed information of 7 maize genotypes. NSS, non-stiff stalk. Maize germplasm information can be found in: www.maizego.org/download/M.pdf.

Maize genotype	Pedigree	Origin	Subpopulations	Adaptation
GY220	AIHO*	China	NSS	Temperate
Shen5003	American Single-cross 3147	China-V	NSS	Temperate
Ji846	Ji63×Mo17	China	NSS	Temperate
835A	U8112×Ye515	China	NSS	Temperate
CIMBL89	(DTPYC9-F143-5-4-1-2-B)-B	CIMMY	Mixed	Tropical/subtropical
CML118	SIYFS3#B1-7-B1-B1#B1	CIMMYT	Mixed	Tropical/subtropical
Zheng35	Unkown	China	Mixed	Temperate

### 2.2 Sample collection and determination

The plant height was measured and sampled at the silking stage (26 July 2021) and maturity (11 September 2021). For nutrient analysis, we sampled and dissected one representative maize plant from each replicate treatment. At the silking stage, samples from the upper leaves, ear leaves, lower leaves, stalks, and roots of the three genotypes were collected separately. At maturity, samples of the upper leaves, ear leaves, lower leaves, grains, bracts, cobs, stalks, and roots of the three genotypes were collected separately. All samples were oven-dried at 105°C for 30 min, dried to a constant weight at 65°C, and the biomass was measured. The samples were ground into a powder and used to measure the P concentration. Roots were carefully separated from the soil and sampled at a depth of 25 cm. To determine the grain yield at maturity, we harvested all grains from 20 maize plants of the three genotypes in each plot.

Total P concentration was measured using an assay based on ammonium molybdate/ascorbic acid (Ames, 1966). The harvest index (HI) was calculated as the ratio of grain dry biomass to total shoot biomass (Aranjuelo et al., 2013) and the P harvest index (PHI) was calculated as the ratio of grain P content to shoot P content (Yang et al., 2022). Grain P utilization efficiency (PUEg) was determined as the ratio of grain yield to total shoot P content. The post-silking P remobilization number of different organs was the difference between the values at maturity and silking. P remobilization fluxes were calculated as the percentage of P in different organs to the grain P content. Micronutrient concentrations in the grains were determined (Cheah et al., 2020) and analyzed using inductively coupled plasma optical emission spectroscopy (ICP-OES; Optima 7300 DV, Perkin Elmer; Wellesley, MA, USA) (Zasoski and Burau, 1977). Phytic acid (PA) concentration was determined as described by Haug and Lantzsch (1983). Zn bioavailability in grains was estimated by two methods: i) using the molar ratio of PA to Zn ([PA]/[Zn]) (Morris and Ellis, 1989), and ii) using a model that calculates the total daily absorbed Zn bioavailability (TAZ, mg Zn day^−1^) (Miller et al., 2007). Fe bioavailability in grains was also estimated by two methods: i) using the molar ratio of PA to Fe ([PA]/[Fe]) and ii) using a factor of 17.2% as the dietary Fe absorption in food, and the index of available Fe (TAF, mg Fe day^−1^) in grains was evaluated (Saha et al., 2017; Su et al., 2018).

### 2.3 Statistical analyses

All statistical analyses were performed using IBM SPSS Statistics software (SPSS, Chicago, IL, USA) with one-way analysis of variance (ANOVA), followed by Duncan’s multiple range test to determine differences (*p <*0.05) among treatment means.

## 3 Results

### 3.1 The growth of three inbred lines CIMBL89, Ji846, and CML118 under different P treatments

**Figure 1:**
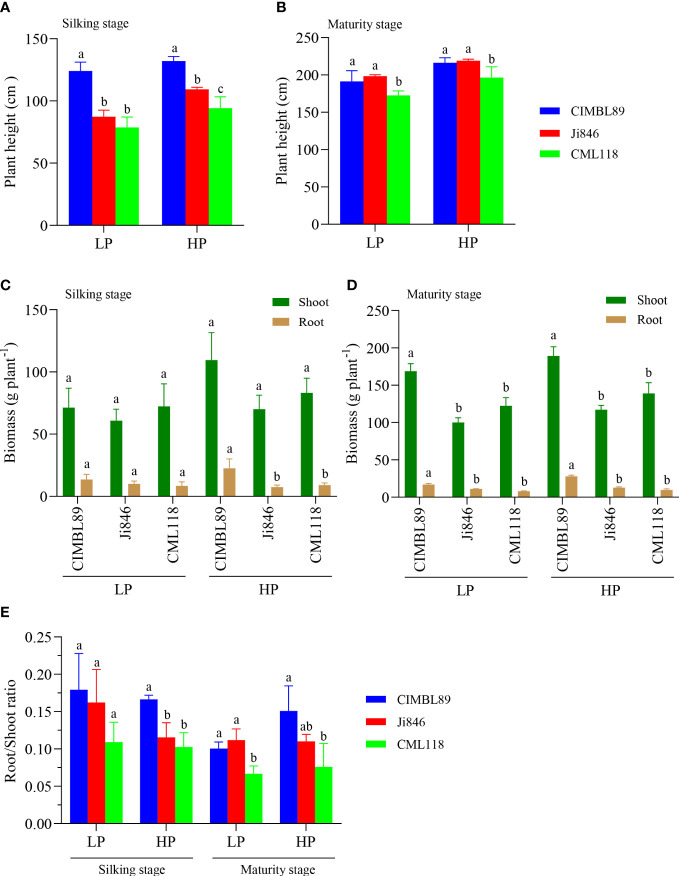
Plant height, plant biomass, and root/shoot ratio of three inbred lines, CIMBL89, Ji846, and CML118, under LP and HP conditions. Plant height at the silking **(A)** and maturity stages **(B)** of three maize genotypes. Plant biomass at the silking **(C)** and maturity stages **(D)** of three maize inbred lines. **(E)** Root/shoot ratio at the silking and maturity stages of three inbred maize lines. Bars indicate the means ± SE of four replicates, and different letters indicate significant differences among maize genotypes at each P supply (*p <*0.05).

The plant height, biomass, and root/shoot ratio of the three inbred lines, CIMBL89, Ji846, and CML118, were estimated at the silking and maturity stages, respectively (**Figure 1**). During the silking stage, CIMBL89 demonstrated significantly greater plant height than Ji846 and CML118 under both LP and HP conditions. Specifically, CIMBL89 showed a significant increase in height of 42% and 58% under LP supply and 21% and 40% under HP supply, respectively (**Figure 1A**). At maturity, there was no significant difference in plant height between CIMBL89 and Ji846, but both were significantly greater than that of CML118 (**Figure 1B**). At the silking stage, there was no difference in shoot biomass between the three inbred maize lines. However, the root biomasses of CIMBL89 under HP was significantly greater than that of Ji846 and CML118, increasing by 204% and 149%, respectively (**Figure 1C**). At maturity, the shoot and root biomass of CIMBL89 were significantly greater than those of Ji846 and CML118 under both LP and HP conditions (**Figure 1D**). We further analyzed the root/shoot ratio of the three inbred lines under different P supplies and found that at the silking stage, the root/shoot ratio of CIMBL89 under HP supply was significantly greater than that of Ji846 and CML118, which increased substantially by 44% and 62%, respectively (**Figure 1E**). At maturity, the root/shoot ratio of CIMBL89 and Ji846 under LP was significantly greater than that of CML118, which increased significantly by 51% and 68%, respectively (**Figure 1E**).

**Figure 2:**
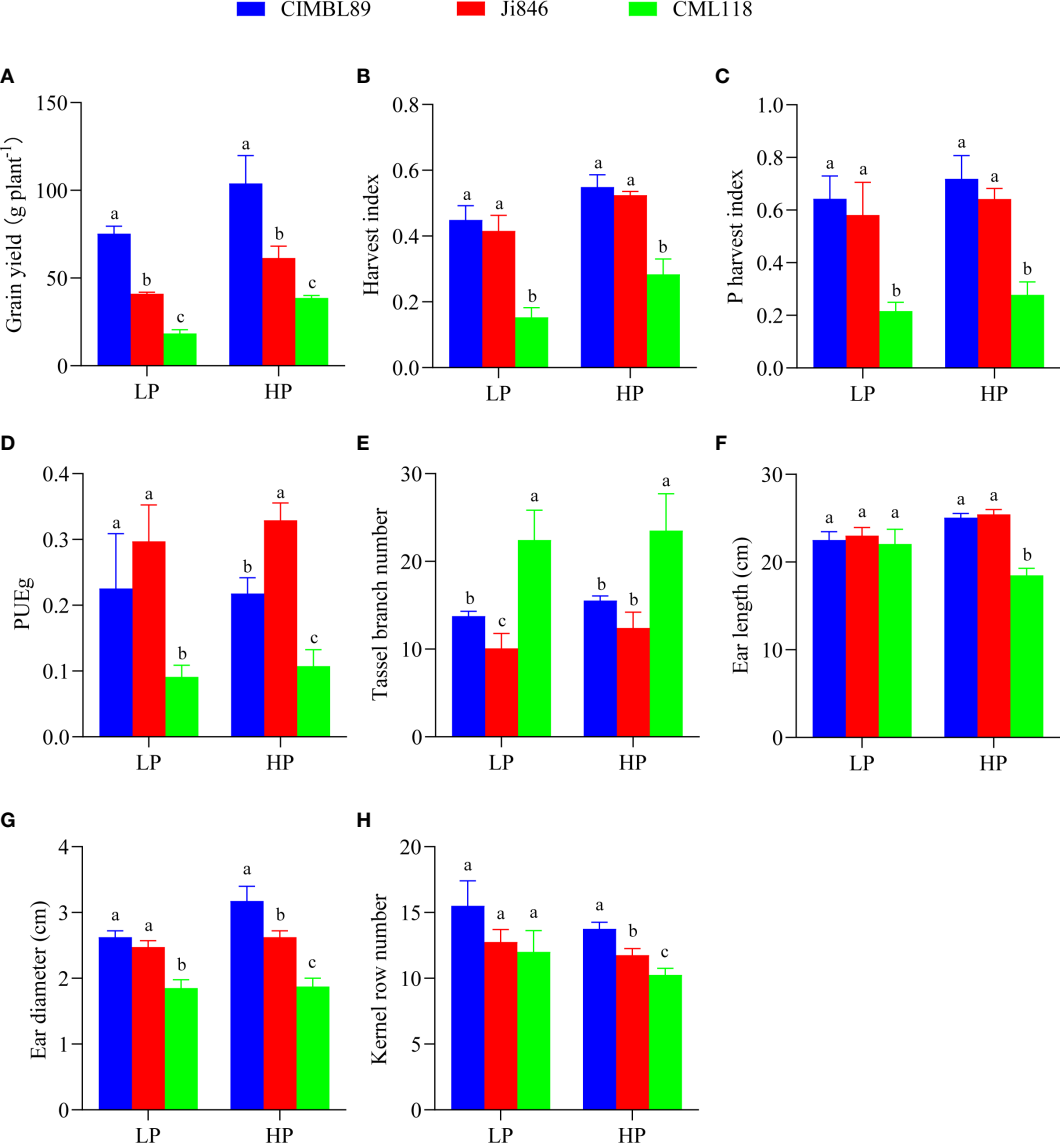
Grain yield **(A)**, harvest index (HI) **(B)**, P harvest index (PHI) **(C)**, grain P utilization efficiency (PUEg) **(D)**, tassel branch number **(E)**, ear length **(F)**, ear diameter **(G)**, and kernel row number **(H)** of three inbred lines, CIMBL89, Ji846, and CML118, under LP and HP conditions. Bars indicate the means ± SE of four replicates, and different letters indicate significant differences among inbred maize lines at each P supply (*p <*0.05).

To further explore the growth of the three inbred maize lines, we evaluated grain yield, harvest index (HI), phosphorus harvest index (PHI), grain P utilization efficiency (PUEg), tassel branch number, ear length, ear diameter, and kernel row number (**Figure 2**). The grain yield of the three genotypes decreased significantly as follows: CIMBL89 > Ji846 > CML118 (**Figure 2A**). The HI of CIMBL89 was greater than that of Ji846 and CML118, increasing by 8% and 194% under LP supply, and 5% and 93% under HP supply, respectively (**Figure 2B**). PHI showed a similar trend among the three genotypes (**Figure 2C**). The PUEg values of CIMBL89 and Ji846 were significantly greater than that of CML118 at the LP supply. At the HP supply, the PUEg of Ji846 was the highest, significantly increasing by 51% and 206% compared with CIMBL89 and CML118, respectively (**Figure 2D**). The tassel branch number of CML118 was significantly greater than that of the other two lines at both LP and HP supply (**Figure 2E**). There was no significant difference in ear length and kernel row number among the three genotypes (**Figures 2F, H**), but the ear diameters of CML118 were 30% and 25% lower than those of CIMBL89 and Ji846, respectively, at LP supply (**Figure 2G**). The ear length, ear diameter, and kernel row number of CML118 were lower than those of CIMBL89 and Ji846 under HP (**Figures 2F–H**).

### 3.2 Total P concentration and total P content in different component organs of three inbred maize lines CIMBL89, Ji846, and CML118, at the silking and maturity stages under different P treatments

**Figure 3:**
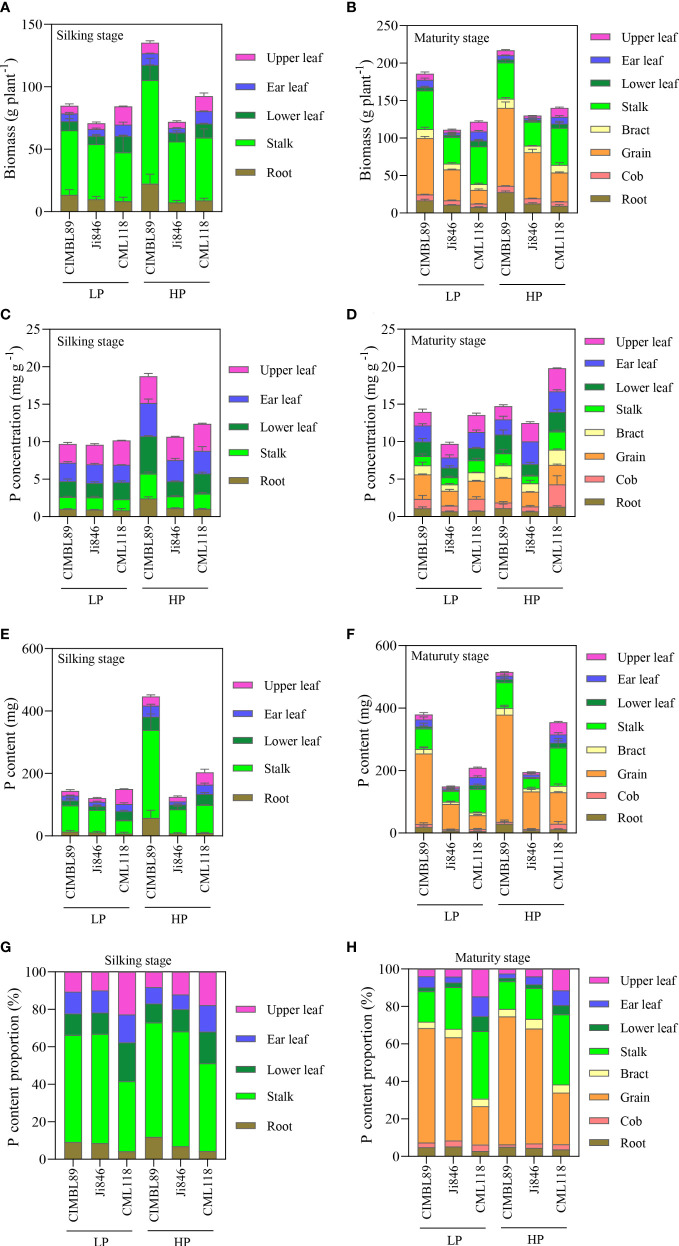
Biomass, total P concentration, total P content, and total P content proportion of different organs of three inbred maize lines, CIMBL89, Ji846, and CML118, under LP and HP conditions. Biomass at the silking **(A)** and maturity stages **(B)** of different organs of the three genotypes. Total P concentration at the silking **(C)** and maturity stages **(D)** of different organs of the three genotypes. Total P content at the silking **(E)** and maturity stages **(F)** of different organs of the three genotypes. Total P content proportion at the silking **(G)** and maturity stages **(H)** of different organs of the three genotypes. Bars indicate the means ± SE of four replicates.

The biomass of different organs of the three inbred lines was significantly different at the silking and maturity stages under different P supplies, as shown in **Figures 3A, B**. At the silking stage, the leaf biomass of CML118 was 87% and 120% greater than that of CIMBL89 and Ji846 under LP supply, and was 11% and 112% greater under HP supply, respectively (**Figure 3A**). At maturity, the leaf biomass of CML118 was greater than that of CIMBL89 and Ji846 under both the LP and HP treatments (**Figure 3B**). Compared with the three genotypes, CML118 had the lowest grain biomass, which was 76% and 55% lower than that of CIMBL89 and Ji846 under LP supply, and was 63% and 37% lower under HP supply, respectively (**Figure 3B**). The root biomass of CIMBL89 was greater than those of Ji846 and CML118 under different P supplies at the silking and maturity stages (**Figures 3A, B**).

The total P concentrations in different organs of the three inbred maize lines are shown in **Figures 3C, D**. At the silking stage, the total P concentration in different organs of the three inbred lines was not significantly different under LP supply (**Figure 3C**). Under HP conditions, the total P concentration in all organs of CIMBL89, except the upper leaves was greater than that of Ji846 and CML118. Specifically, the total P concentration was 57% and 47% greater in ear leaves, 152% and 87% greater in lower leaves, 108% and 63% greater in stalks, and was 114% and 133% greater in roots than that in Ji846 and CML118, respectively (**Figure 3C**). At maturity, CIMBL89 had the greatest grain P concentration, which was 66% and 37% greater than that of Ji846 and CML118 at LP supply, and 69% and 26% greater at HP supply (**Figure 3D**). Compared with CIMBL89 and Ji846, CML118 showed higher P concentrations in cobs and stalks. In cobs, the P concentration of CML118 was 29% and 111% greater than that of CIMBL89 and Ji846 at LP supply, and was 300% and 346% greater at HP supply (**Figure 3D**). In stalks, the P concentration of CML118 was 31% and 74% greater than that of CIMBL89 and Ji846 at LP supply, and 49% and 138% greater at HP supply, respectively (**Figure 3D**). Moreover, the P concentrations in the ear leaves of CIMBL89 were 33% and 26% lower than those of Ji846 and CML118 under HP supply, respectively (**Figure 3D**).

The three genotypes showed different trends in P uptake under HP and LP treatments (**Figures 3E, F**). Regarding P-sufficient supply, there was an increase in P contents from 446.9 mg plant^−1^ to 515.7 mg plant^−1^ for CIMBL89, from 125.4 mg plant^−1^ to 195.4 mg plant^−1^ for Ji846, and from 203.5 mg plant^−1^ to 354.6 mg plant^−1^ for CML118, between anthesis and maturity, as shown in **Figures 3E, F**. Interestingly, CIMBL89 showed strikingly increased whole P content than that of Ji846 and CML118, ranging from 144.4 to 380 mg P plant^−1^, from 121.1 mg P plant^−1^ to 149.1 mg P plant^−1^ and from 150.1 mg P plant^−1^ to 208.5 mg P plant^−1^ from anthesis to maturity at LP supply, respectively (**Figures 3E, F**).

The three inbred lines had different P distribution patterns, as shown in **Figures 3G, H**. At the silking stage, the proportion of P in the leaves of CML118 was 59% and 49% under LP and HP conditions, respectively, which was significantly greater than that of CIMBL89 and Ji846, whereas the proportion of P in stalks and roots followed the opposite trend (**Figure 3G**). At maturity, the proportions of P in the leaves and stalks of CML118 were significantly greater than those of CIMBL89 and Ji846 under both LP and HP supply, whereas the proportion of P in grains was 66% and 62% lower than that of CIMBL89 and Ji846 under LP supply, and 61% and 56% lower under HP supply, respectively (**Figure 3H**). Under P-sufficient supply, CIMBL89 and Ji846 allocated more than 68% and 61% of the total P to the grain, respectively whereas CML118 allocated only 27% (**Figure 3H**).

### 3.3 Post-silking P fluxes of three inbred maize lines, CIMBL89, Ji846, and CML118, at whole plant level under both LP and HP supply

**Figure 4:**
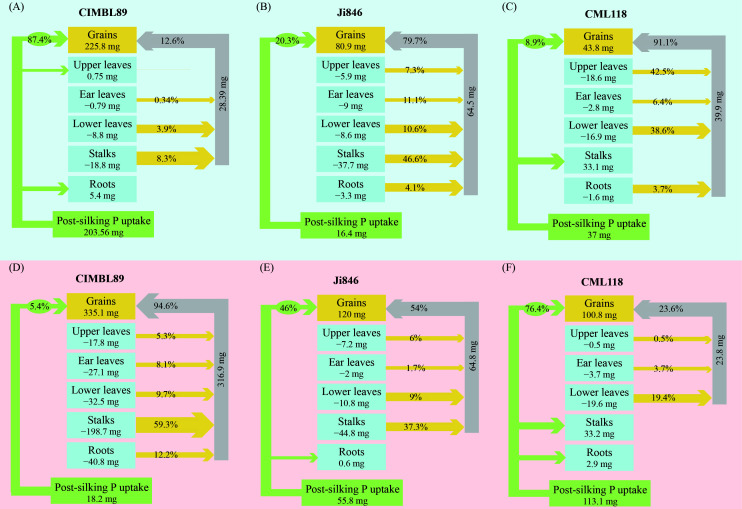
Phosphorus uptake and remobilization to grains during the post-silking period of three inbred maize lines, CIMBL89, Ji846, and CML118, under LP **(A–C)** and HP **(D–F)** conditions. The values in each box represent the amount of post-silking P remobilization from different organs to the grains, with the unit of measurement being mg. The percentages on the green arrows indicate the amount of P uptake from the soil after silking. The percentages on the yellow arrows represent the contribution rate of different organs to the overall P content in the grains. Finally, the percentages on the gray arrows indicate the total contribution rate of all organs to the P content in the grains.

As shown in **Figure 4**, there were significant differences in P uptake and remobilization across the different organs of the three inbred maize lines (CIMBL89, Ji846, and CML118) after silking under both LP and HP conditions. Under P-deficient conditions, P uptake was significantly increased in CIMBL89, accounting for 54% of the total P in mature plants and contributing to 87% of P in the grain (**Figure 4A**). In contrast, for Ji846 and CML118, approximately 80% and 90% of grain P, came from the P remobilization of plant organs. Moreover, the P absorbed by CML118 was allocated to grains and stalks. Under HP conditions, almost all (95%) of the P in the grains of CIMBL89 came from the remobilization of P in various organs (roots, stalks, lower leaves, ear leaves, and upper leaves). Among them, stalks contributed 59% of the grain P (**Figure 4D**). In contrast, approximately half of the grain P in Ji846 came from the P remobilization of plant organs (**Figures 4B, E**). Approximately quarter of the grain P in CML118 came from the remobilization of P in the lower leaves, ear leaves, and upper leaves, whereas the remainder came from the uptake of P after silking (**Figure 4F**).

### 3.4 Micronutrients and phytic acid concentrations in grains of three inbred lines, CIMBL89, Ji846, and CML118, under different P supplies

**Figure 5:**
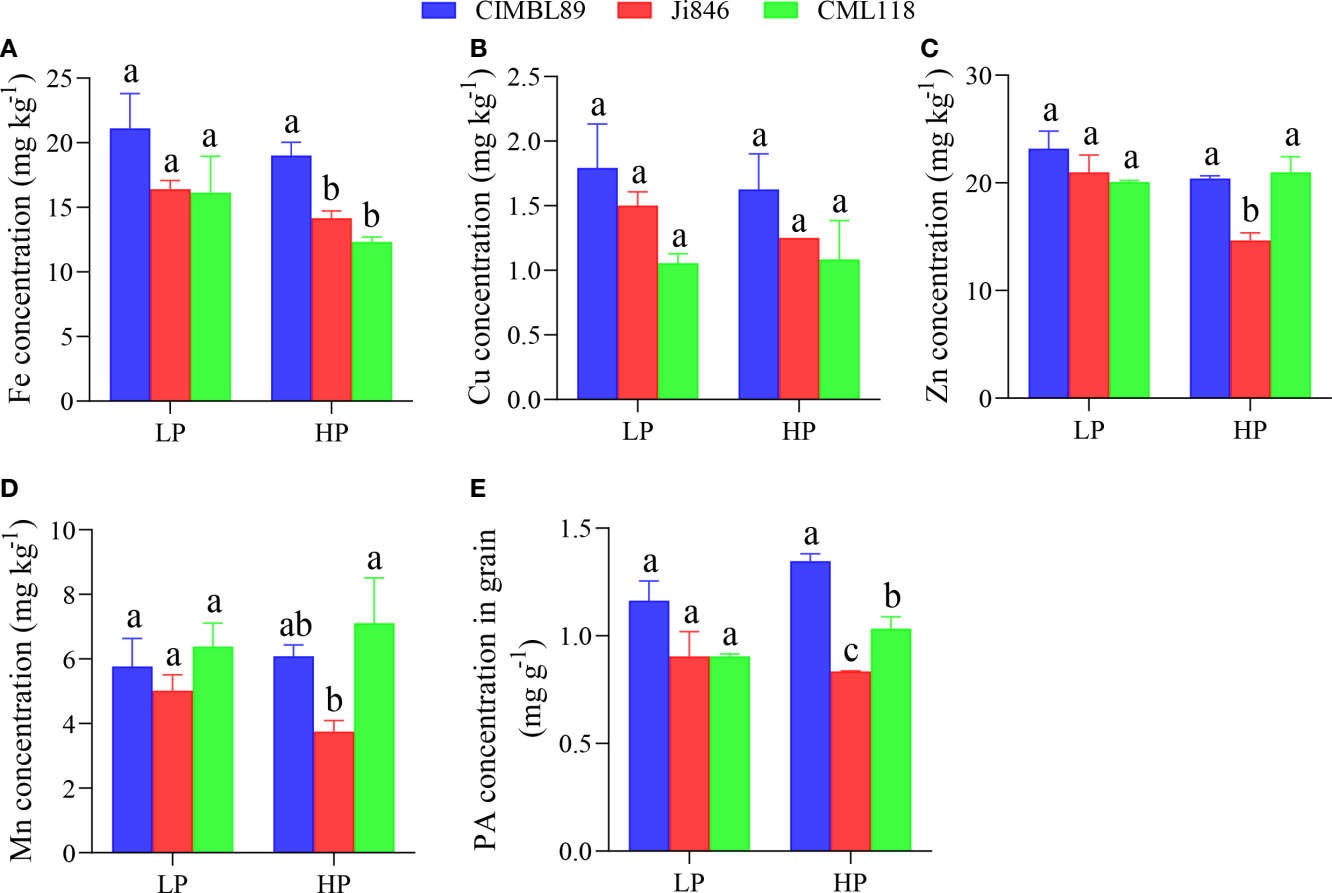
Micronutrients and phytic acid (PA) in grains of three inbred lines, CIMBL89, Ji846, and CML118, under LP and HP conditions. Fe **(A)**, Cu **(B)**, Zn **(C)**, Mn **(D)**, and PA **(E)** concentrations in grains of three maize genotypes. Bars indicate the means ± SE of four replicates, and different letters indicate the significant differences among maize genotypes at each P supply (*p <*0.05).

Four micronutrients, Fe, Cu, Zn, and Mn, were determined in three inbred lines. There was no significant difference in micronutrient concentrations in the grains of the three inbred lines under LP conditions. The grain Fe concentration (mg kg^−1^) was significantly lower in the HP-treated plants than in the LP-treated plants, irrespective of the maize genotype (**Figure 5A**). Under HP conditions, the concentration of Fe in the grains of CIMBL89 was significantly higher than that in Ji846 and CML118 (**Figure 5A**). Additionally, the concentrations of Zn and Mn in the Ji846 grains were significantly lower than those in the CIMBL89 and CML118 grains (**Figures 5C, D**).

The PA concentration in the grains was then measured. There were no significant differences in PA concentrations among the three inbred lines under LP conditions. However, under HP conditions, the PA concentration among the genotypes followed the order: CIMBL89 > CML118 > Ji846 (**Figure 5E**).

### 3.5 Zn and Fe bioavailability in grains of three inbred maize lines, CIMBL89, Ji846, and CML118 at both LP and HP supply

**Figure 6:**
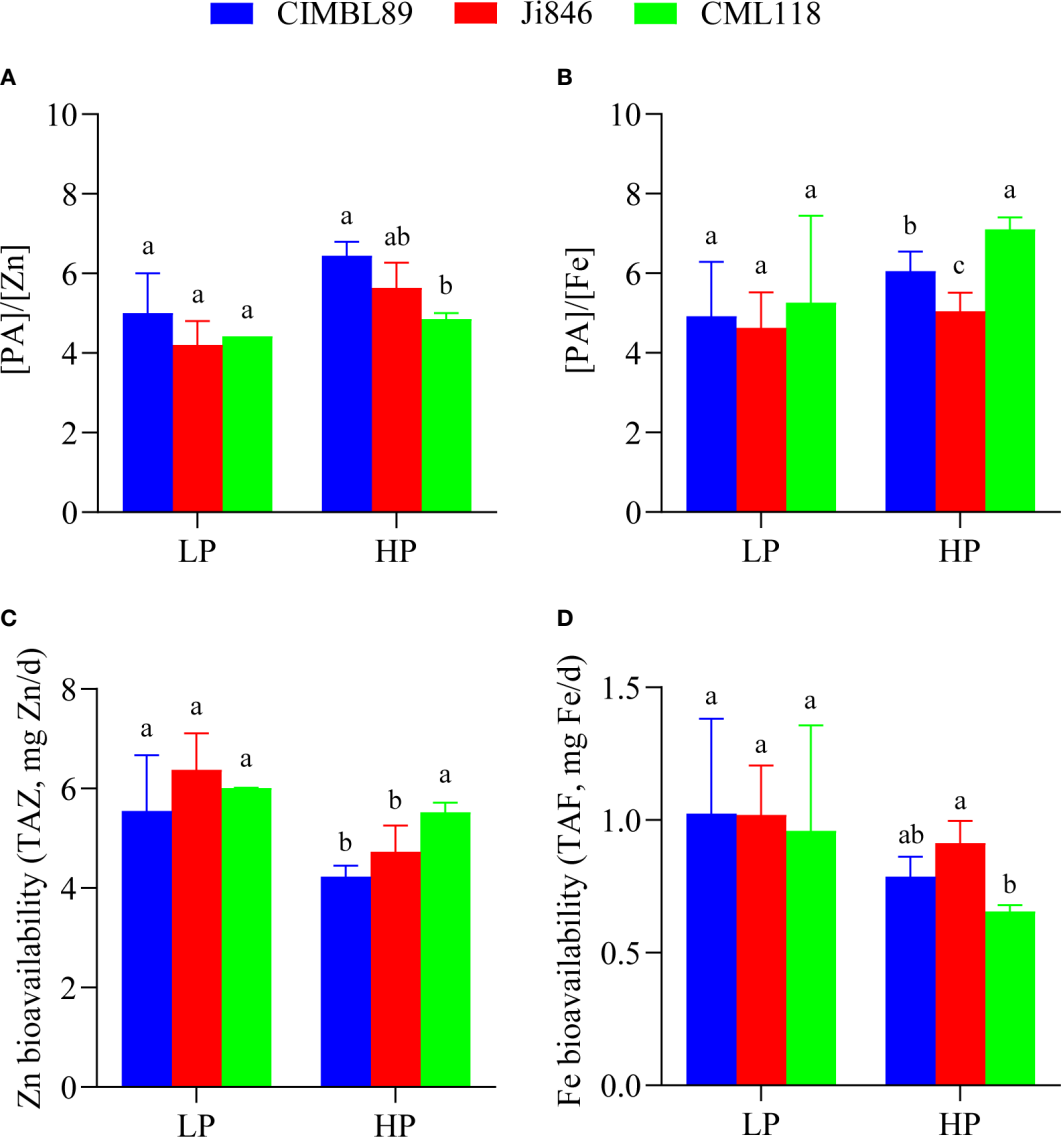
Alterations in [PA]/[Zn] **(A)**, [PA]/[Fe] **(B)**, and mineral (Zn, Fe) bioavailability (TAZ **(C)**, TAF **(D)**) were affected under LP and HP conditions. Bars indicate the means ± SE of four replicates, and different letters indicate the significant differences among maize genotypes at each P supply (*p <*0.05).

The values of [PA]/[Zn] and [PA]/[Fe] in maize grains were affected by different P supplies, with higher [PA]/[Zn] and [PA]/[Fe] values under HP conditions than under LP conditions (**Figures 6A, B**). Under a P-sufficient supply, the [PA]/[Zn] and [PA]/[Fe] in the grains of CIMBL89 were 29% and 23% greater, respectively, than those in the LP supply (**Figures 6A, B**). We also evaluated the availability of Zn and Fe (TAZ and TAF) in maize grains and found that higher TAZ and TAF values were associated with LP levels (**Figures 6C, D**). Moreover, we observed that higher TAZ and TAF levels in humans were associated with lower values of [PA]/[Zn] and [PA]/[Fe].

## 4 Discussion

### 4.1 Root architecture and activity affect post-silking P uptake at LP supply

Roots play a crucial role in maintaining plant productivity, and one strategies to efficiently acquire efficiently more nutrients is to adjust root architecture and morphology for greater exploration in the soil (Niu et al., 2013; White et al., 2013). The root biomass of CIMBL89 was 35% and 61% greater than that of Ji846 and CML118, respectively, under LP conditions at the silking stage (**Figures 3A, B**). Good root architecture is crucial for the acquisition of nutrients by maize, and a larger root architecture is more conducive to P acquisition (Niu et al., 2013). We found that under LP conditions, maize genotypes with shorter lateral roots had 89% greater P acquisition than those with few longer lateral roots. Moreover, these genotypes have 81% greater root length density in the topsoil (0–20 cm) and 14% greater grain yield (Jia et al., 2018). In water hyacinth (*Eichhornia crassipes*), the lateral root length and density under LP supply were 2.4-fold and 2.0-fold, respectively and the lateral root diameter was 20% lower than that under HP supply (Xie and Yu, 2003). Genotypes with higher lateral rooting activity had greater P acquisition efficiency, biomass, and relative growth rates under LP conditions, and lateral root extension required lower metabolic costs (nutrient investments) than other root types (Zhu and Lynch, 2004). Genotypes with higher lateral branching density in the topsoil and lower lateral branching density in the subsoil may have greater adaptation to soils with LP availability (Postma et al., 2014). Soil exploration plays an important role in nutrient acquisition, but the efficiency of soil exploration is limited by the amount of carbon and nutrients used by roots (metabolic costs) (Lynch et al., 2005; Galindo-Castañeda et al., 2018). Our results showed that the post-silking P uptake of CIMBL89 was 203.56 mg, which was 12-fold and 5-fold higher than that of Ji846 and CML118, respectively (**Figure 4A**). Moreover, the root biomass of CIMBL89 increased by 26% from silking to maturity (**Figures 3A, B**). These findings suggest that the roots of CIMBL89 can efficiently obtain P through soil exploration under LP after silking, thereby maintaining normal growth and metabolism.

### 4.2 Post-silking P uptake and remobilization and grain formation in maize

Post-silking dry matter accumulation, nutrient accumulation, and nutrient remobilization are closely related to the levels of N, P, and K (Ray et al., 2020). Differences in post-silking P uptake and grain yield exist among different inbred lines, particularly under LP conditions (Zhang et al., 2013). Our results showed that post-silking P uptake and remobilization of CIMBL89 were significantly affected by different P supplies. The uptake of P by roots accounted for 87% of the grain P content in the LP supply, while the remobilization of P by different organs accounted for 95% of grain P content in the HP supply (**Figures 4A, D**). Compared to the P-sufficient environment, the roots of CIMBL89 activate and acquire more P to sustain grain P accumulation after silking during P deficiency. Given the urgent need to improve phosphorus acquisition efficiency from the soil and reduce the input of P fertilizer under P-deficient conditions, crop genetic breeding for efficient P acquisition is crucial (Richardson et al., 2009). High genotypic variability with different P-acquisition strategies has been observed in Chilean lowland quinoa accessions grown under low available-P conditions, which may be mainly due to root architecture and morphology rather than biochemical activity (de Souza Campos et al., 2022). Our results showed that CIMBL89 had extremely low P recycling efficiency under LP supply, suggesting that this inbred line may be more tolerant to P deficiency.

Phosphorus remobilization is a key factor controlling PUE and yield formation in agroecosystems, where the strength and size of sink organs play an important role *via* feedback regulation (Wang and Ning, 2019). Grain P accumulation in Ji846 and CML118 depended mainly on P remobilization in various organs under LP conditions. Julia et al. (2016) quantified the uptake, distribution, and redistribution of P to rice grains during the grain filling stage using isotope tracer technology, while pointed out that grains were the main P pool and grain P accumulation was mainly due to the remobilization of P acquired before flowering and stored in vegetative organs. The remobilization of P from vegetative organs to grains plays an important role in the global P cycle (Jeong et al., 2017). Growth and development of grains and grain P accumulation depend mainly on the recycling of P in vegetative organs (source tissues), especially under P-deficient conditions during the grain-filling stage (Marschner et al., 1996). Under HP conditions, CIMBL89 exhibited almost no further P uptake after silking, whereas the P uptake of CML118 made the dominant contribution to grain P (76%), with 68% flowing to the grains and 29% to the stalks (**Figures 4D, F**). In contrast to CIMBL89 and Ji846, the P in stalks of CML118 was not involved in export and reuse, whereas those of CIMBL89 and Ji846 contributed 59% and 37% to the grains, respectively (**Figures 4D–F**). Our findings suggest that under a P-sufficient supply, CIMBL89 can transfer and reuse P stored during vegetative growth to contribute to grain formation, thereby avoiding the luxury absorption of P from the soil. In general, excessive nutrient input can lead to extravagant absorption and nutrient depletion of nutrients by crops in the soil (Soder and Stout, 2003; Herencia et al., 2011). In intercropping systems, grain P concentration in maize was significantly increased by 8%–26%, likely due to extravagant P absorption by roots and superior acquisition of interspecific P accompanied by a lower efficiency of internal P remobilization (Xia et al., 2019). The differences in grain yield among the three maize inbred lines under LP and HP conditions may be attributed to their distinct strategies of P flux.

### 4.3 Modulation of maize ear and tassel development at the molecular level to improve yield and provide breeding potential

Maize grain yield can be described by several important agronomic traits such as effective ear number, kernels per ear, kernel size, and kernel weight (Chen et al., 2016). Kernel row number is an important component of measuring the number of kernels per ear and exhibits higher heritability (Dhillon and Singh, 1977), which mainly reflected in three traits: kernel length, kernel width, and kernel thickness (Wang et al., 2020b). Our results showed that CML118 had the smallest ear length, ear diameter, and reduced kernel row number, all of which negatively affected grain yield per plant at HP supply (**Figures 2F–H**). Bommert et al. (2013) have pointed out that the continuous domestication of maize and other cereal crops has had profound impact on the development of agriculture and the establishment of human civilization. Modern maize varieties can produce approximately six to 18 more rows per ear than their ancestor teosinte, thereby greatly increasing grain yield potential. The kernel row number per ear varied to a certain extent among the different maize varieties. Therefore, exploring the causative genes closely related to these traits during the development of the three maize genotypes while precisely determining the sampling period is crucial.

Generally, the relatively complex genetic structure of grain yield-related traits is determined by multiple genes, which are referred to as quantitative trait loci (QTL) (Baye et al., 2022). Many QTLs associated with grain yield have been identified in the maize genome related to kernel weight including qGW4.05 (Chen et al., 2016), qGW1.05 (Zhou et al., 2017), qhkw5-3 (Li et al., 2019), qKW9 (Huang et al., 2020); Those related to kernel length including qKL-2 (Wang et al., 2020b), qKL1.07 (Qin et al., 2016), qKL9 (Gong et al., 2021); Those related to kernel width including qKW-1 (Wang et al., 2020b), qKW-2 (Wang et al., 2020b), qKW7 (Li et al., 2016), qKW9.2 (Raihan et al., 2016); Those related to kernel row number including qKRN1 (Wang et al., 2019b), qKRN2 (Liu et al., 2016), qKRN5.04 (An et al., 2022), qKRN5 (Bommert et al., 2005), qKRN5b (Shen et al., 2019), qKRN4 (Taguchi-Shiobara et al., 2001; Liu et al., 2015), qKRN8 (Han et al., 2020), qkrnw4 (Nie et al., 2019). The phenotypic differences observed among the three inbred lines (CIMBL89, Ji846, and CML118) can be used to investigate and screen differentially expressed genes that affect ear and grain development-related traits in a targeted manner, allowing for quick and efficient identification of major and minor genes.

Tassel branch number is a quantitative trait controlled by major genes and polygenes, and many QTLs have been mapped across different environments (Brewbaker, 2015; Chen et al., 2017). Tassel branch number, an important agronomic trait, has been observed to compete with the ear for sunlight and nutrients, ultimately leading to reduced maize production (Brown et al., 2011; Qin et al., 2021). A maize genotype with a small tassel has a greater yield, especially under higher planting density conditions (Hunter et al., 1969). Under the premise of guaranteeing the amount and duration of pollen scattering, it is feasible to improve maize yield potential by completely or partially removing the tassel by physical means or by the targeted regulation of genes related to tassel structure (Upadyayula et al., 2006). In our study, we observed that the tassel branch number of CML118 was significantly increased by 49% and 107% with LP supply and by 32% and 74% with HP supply compared to CIMBL89 and Ji846, respectively (**Figure 2E**). As a result, the grain yield of CML118 was significantly lower than that of the other two lines, possibly because of the increased number of tassel branches.

### 4.4 The micronutrients in maize grains and human nutrition and health

In addition, our study examined and compared the micronutrient content and Zn/Fe bioavailability in maize grains (**Figures 5**, **6**) as part of efforts to prevent micronutrient deficiencies, which is one of the three main areas that require implementation in nutrition-sensitive agricultural production. In agroecosystems, micronutrients refer to elements at low concentrations (usually expressed in mg kg^−1^ or less) (He et al., 2005). The hygienic standard for Cu limits in food, such as cereals, is ≤10 mg kg^−1^. Our results indicate that the Cu concentration in maize grains was within the safety standard range, measuring less than 2.0 mg kg^−1^ and with no significant difference observed at both LP and HP supply (**Figure 5B**). As an essential micronutrient for human health, Zn has anti-oxidative stress and anti-inflammatory effects and is involved in numerous biochemical pathways related to the growth, metabolism, and immunity of human cells (Prasad, 2014; Choi et al., 2018; Tamura, 2021). Inadequate dietary intake of Zn is one of the important causes of Zn deficiency in humans, and individuals with severe Zn deficiency exhibit growth retardation, immune system dysfunction, nervous system diseases, and other chronic diseases such as cancer (Devirgiliis et al., 2007; Szewczyk, 2013). Within the limits of the National Food (cereals) Safety Standard (≤50 mg kg^−1^), reasonable Zn supplementation is beneficial to ensure human micronutrient nutrition and human health. As maize grain is the main component of human consumption, its internal Zn nutrition can be a reference factor to measure the nutritional value of different maize inbred lines. In our study, there was no difference in grain Zn concentration among three inbred lines at LP supply. However, at HP supply, the grain Zn concentration of Ji846 was significantly lower than that of CIMBL89 and CML118 (**Figure 5C**). The concentration of Fe, Zn, and Mn in Ji846 seeds was lower when grown under HP compared to low pressure (LP). However, it is interesting to note that the seed P concentration of Ji846 was not affected by P treatments and was the lowest among the inbred lines studied (**Supplementary Figure 1**). Therefore, it is possible that the decrease in Fe, Zn, and Mn concentration was due to a “dilution effect” caused by the larger seed biomass of Ji846 grown under HP compared to LP. Further analysis revealed that the Zn bioavailability in the grains of CML118 was significantly higher than that of the other two lines at HP supply (**Figures 6A, C**). As a result, CML118 is better able to prevent or overcome dietary Zn deficiency in humans, a global health issue that particularly affects developing countries and already impacts approximately 2 billion people (Pan et al., 2017).

Fe-deficiency anemia is another global public health problem and one of the main causes of physical defects/diseases that people have suffered for many years, affecting over 1.2 billion people worldwide, particularly women (Stevens et al., 2013; Camaschella, 2019; Benson et al., 2021). Iron deficiency reduces the function of Fe-containing enzymes, which affect substance and energy metabolism as well as the synthesis of hemoglobin. This results in Fe deficiency anemia, which interferes with the normal growth and differentiation of cells, the synthesis of neurotransmitters, and reduces the body’s immunity and cardiopulmonary function, among other effects (Drakesmith and Prentice, 2012; Litton and Lim, 2019; Benson et al., 2021). A moderate increase in the intake of Fe in our daily diet is the preferred measure to meet the body’s physiological requirements for Fe and to effectively avoid the occurrence of Fe-deficiency diseases, although not all Fe from the diet is utilized by humans (Diego Quintaes et al., 2017; Benson et al., 2021). Increasing the relative Fe bioavailability of the edible fraction of cereal crops appears to be more cost-effective due to the growing demand for food (Diego Quintaes et al., 2017). Our results showed that the grain Fe concentration of CIMBL89 was 29% and 31% greater than that of Ji846 and CML118 at LP supply, respectively, although this difference was not significant. However, at HP supply, the grain Fe concentration of CIMBL89 increased significantly by 34% and 54% compared to Ji846 and CML118, respectively (**Figure 5A**). Further analysis indicated that CML118 had the lowest Fe bioavailability (**Figures 6B, D**). Therefore, CIMBL89 and Ji846 have the potential to support the World Health Organization’s goal of reducing the prevalence of anemia in women of reproductive age by 50% between 2010 and 2025 (Young, 2018; Benson et al., 2021).

## 5 Conclusions

Our study revealed significant differences among the three maize inbred lines (CIMBL89, Ji846, and CML118) in terms of post-silking P uptake and remobilization to different organs, including grains, under both LP and HP conditions. Under LP conditions, the majority of grain P (87%) in CIMBL89 originated from post-silking P uptake, with only 13% contributed by P remobilization. In contrast, grain P in Ji846 and CML118 primarily resulted from P remobilization. This suggests that CIMBL89 has a greater soil P demand during the post-silking period and could exacerbate the depletion or reduction of P resources in P-deficient soils. However, under HP conditions, 95% of the grain P in CIMBL89 originated from the remobilization of P in the roots, stalks, and leaves. Therefore, CIMBL89 exhibited a higher recycling efficiency of P throughout the post-silking period at HP supply.

In comparison to CIMBL89 and Ji846, CML118 exhibited significantly reduced grain yield, HI, PHI, and PUEg, as well as a smaller ear diameter, while the tassel branch number was significantly increased under both LP and HP conditions. Moreover, CML118’s ear length and kernel row number were significantly lower than those of CIMBL89 and Ji846 at HP supply. As such, these inbred lines could prove useful for genetic effect analysis and/or fine mapping of quantitative trait loci (QTLs) concerning ear development and grain formation. Given that maize grain constitutes the primary component of human dietary intake, improving the concentration and bioavailability of micronutrients as well as the phytic acid content of maize grains is crucial to meeting the physiological needs of the human body. Doing so would promote human health by reducing micronutrient deficiencies and minimizing potential environmental risks.

## Data availability statement

The original contributions presented in the study are included in the article/**Supplementary Material**. Further inquiries can be directed to the corresponding author.

## Author contributions

YS, YH, and LC designed the study. YS, YH, ZX, and JZ performed experiments. YS and YH analyzed data. YS, YH, and LC wrote the manuscript. YS, JS and LC revised the manuscript. All authors contributed to the article and approved the submitted version.

## Supplementary material

Supplementary Figure 1 Seed phosphorus (P) concentration of 7 maize genotypes under HP **(A)** and LP **(B)** conditions. Bars indicate means ± SE of four replicates, and different letters indicate significant differences among maize inbred lines (*p* < 0.05).
