# Supplementary figures and images for: Phosphorus partitioning contribute to phosphorus use efficiency during grain filling in Zea mays
Source: Front Plant Sci. 2023 Jul 4;14:1223532. doi: 10.3389/fpls.2023.1223532 (PMC10352663; doi:10.3389/fpls.2023.1223532)

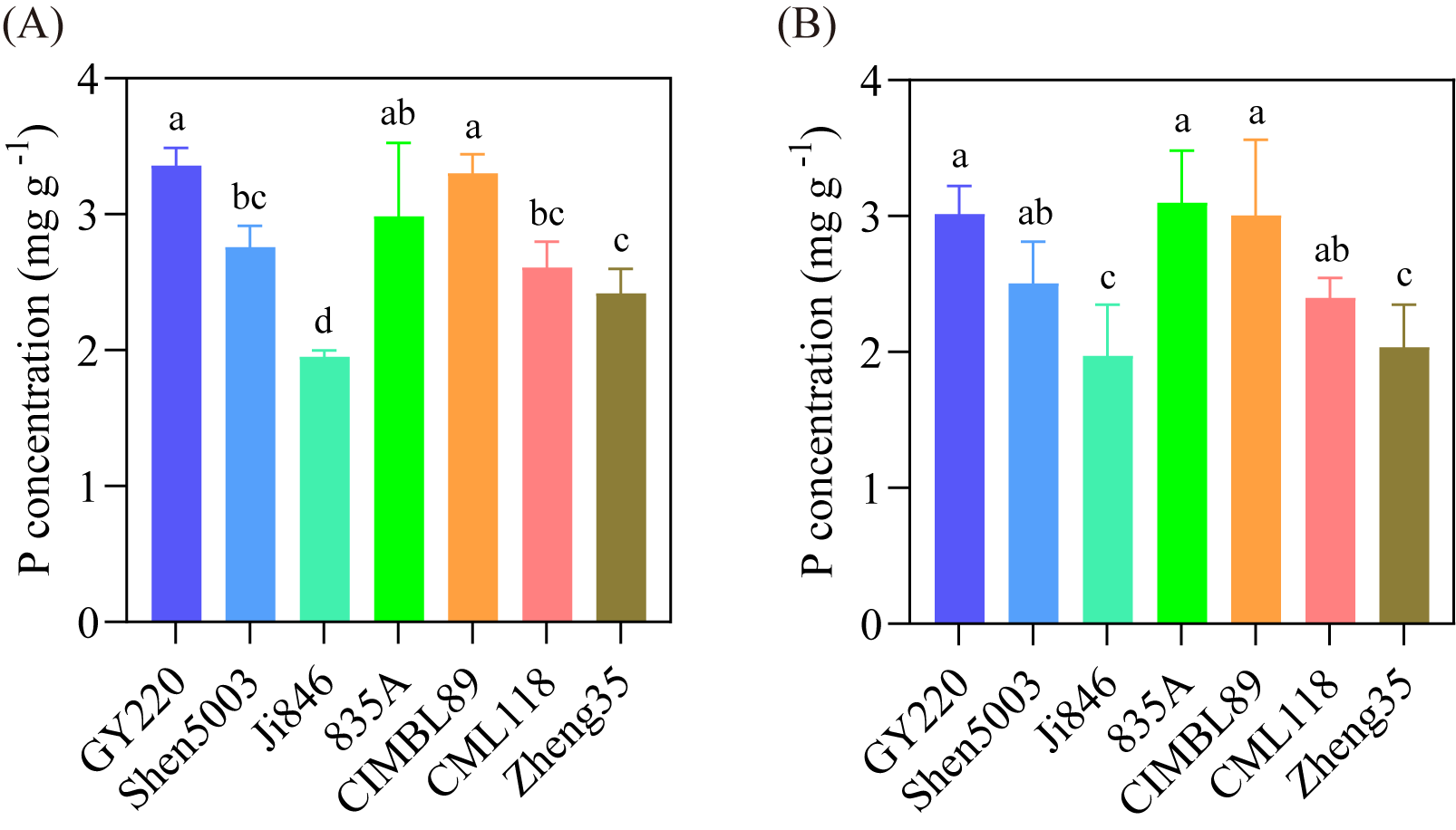

Supplement: Supplementary Figure 1 — Seed phosphorus (P) concentration of 7 maize genotypes under HP (A) and LP (B) conditions. Bars indicate means ± SE of four replicates, and different letters indicate significant differences among maize inbred lines (p < 0.05). [file Image_1.tif]
